# Report on Practical Medicine

**Published:** 1872-10

**Authors:** 


*Report on Practical Medicine made to the Illinois State Medical Society, at the Annual Meeting in May* 1872. By T. D. Washburn, M. D., Hillsboro, Ill.

The prevalent diseases and general conditions of Country and Climate are desribed, in manner to give much instruction as to the causes and nature of the diseases of the state. There is much in the report worth re-produciug. We have space for one paragraph upon practical medicine only.

"Within the last few years, quite a number of active agents have been brought before the profession, and extensively introduced by *reputable* firms, who had spared no pains in advertising them, and giving them all sorts of seductive attractions in the form of syrups, elixirs, capsules, dragées, granules, lozenges, etc.; the popular reputation which certain articles possess in a given class of cases, as Buchu in kidney and bladder affections, strychnia and phosphorus in impaired nerve force, iodoform and iron as an alterative tonic and anti-neuralgic, have given them much favor with the profession. At the same time various concentrated alkaloids and resinoids, which have taken the place of the crude roots, barks, and leaves (under which Thompsonianism sprang into life) have been adopted in practice, and enjoy the confidence of the medical world; but with these have appeared still another class, in magnitude *infinitesimal*, in action *herculean;* strange to say they are alike used by *all* the *schools* and *systems* of medicine, not excepting homœopathy; I allude to the use of hypodermic injections, chloroform, chloral, curare, Calabar bean, cannibis Indica, croton oil, aconite, atropia, amyl, or hydramyl, gelsiminum, strychnia, *et id genus omne; all*, I believe, of *inoffensive* and *harmless* origin, the *vegetable* kingdom, (which thinketh no evil, and doeth no evil with eclectics), but somehow wonderfully increasing the columns of mortality, and giving special activity to the *undertaker*.

I enter no protest to their use. I believe them, in skilful hands, the very boon of Heaven to suffering humanity, but I venture a word of caution against their *abuse*, and earnestly urge upon all, *moderation*, *carefulness* and *discrimination*."

